# Peroxisome Proliferator-Activated Receptor *β*/*δ* in the Brain: Facts and Hypothesis

**DOI:** 10.1155/2008/780452

**Published:** 2008-11-09

**Authors:** M. G. Hall, Laure Quignodon, Béatrice Desvergne

**Affiliations:** Center of Integrative Genomics, Faculty of Biology and Medicine, University of Lausanne, 1015 Lausanne, Switzerland

## Abstract

peroxisome proliferator-activated receptors (PPARs) are nuclear receptors acting as lipid sensors. Besides its metabolic activity in peripheral organs, the PPAR beta/delta isotype is highly expressed in the brain and its deletion in mice induces a brain developmental defect. Nevertheless, exploration of PPAR*β* action in the central nervous system remains sketchy. The lipid content alteration observed in PPAR*β* null brains and the positive action of PPAR*β* agonists on oligodendrocyte differentiation, a process characterized by lipid accumulation, suggest that PPAR*β* acts on the fatty acids and/or cholesterol metabolisms in the brain. PPAR*β* could also regulate central inflammation and antioxidant mechanisms in the damaged brain. Even if not fully understood, the neuroprotective effect of PPAR*β* agonists highlights their potential benefit to treat various acute or chronic neurological disorders. In this perspective, we need to better understand the basic function of PPAR*β* in the brain. This review proposes different leads for future researches.

## 1. INTRODUCTION

Nuclear receptors (NRs) represent the largest family of transcription factors [1]. Up to today, 48 nuclear receptors have been described in humans and 49 in mice [2]. Most of them are ligand-dependent receptors; their specific ligands correspond to a diversity of hormones, steroids, fat-soluble vitamins, fatty acids, oxysterols, bile acids, and dietary lipids [3]. This broad range of ligand diversity and capacity to regulate gene expression makes the NRs key regulators of many pathways involved in reproduction, metabolism, and development. The central nervous system (CNS) expresses nearly all the NRs [2], but for most of them we are still missing in-depth knowledge of their role in brain development, cognition, behavior, and neurological or psychiatric disorders [4]. Among the NR superfamily, the peroxisome proliferator-activated receptors (PPARs), which are described as lipid sensors, are the focus of intense interest, particularly in the context of metabolic disorders and the associated search for new therapies. Amazingly, in addition to its metabolic activity in peripheral organs [5], the PPAR beta/delta isotype is highly expressed in the brain [6] and its deletion in mice is associated with a brain developmental defect [7].

In vertebrates, the PPAR family is composed of three different isotypes. They are known as PPAR*α* (NR1C1), PPAR*β* (NR1C2) also named PPAR*δ* , and PPAR*γ* (NR1C3) [8, 9]. PPARs regulate whole body metabolism in response to dietary lipid intake, by directing their subsequent metabolism and storage. Among their endogenous ligands are poly-unsaturated fatty acids and lipid derivatives such as eicosanoids. However, the search for specific ligands interacting with the three individual receptors of the family has been difficult, owing to their relatively low affinity interactions and broad ligand specificity. PPAR: retinoid-X-receptor (RXR) heterodimers represent the functional entities and bind to conserved regulatory DNA elements and termed peroxisome proliferator response elements (PPREs). PPREs correspond to a repetition of two hexamers, derived from the AGGTCA consensus motif, separated by one nucleotide, and we still understand little on the binding selectivity of the three PPAR isotypes according to the nucleotide sequence of these response elements. PPAR-mediated transcriptional activity is a multistep process. In the absence of ligands, PPAR is associated with corepressors. Upon ligand binding, they are replaced by coactivators, which recruit the basal transcriptional machinery. Thus, PPAR transcriptional activity is depending on a combination of ligand availability, RXR expression, and numerous cofactor interactions. This complexity together with a relatively specific tissue expression of PPAR*α* and PPAR*γ* contributes to the selective PPAR isotype activity.

PPAR*β* is an intriguing member of the PPAR family. It presents a fairly ubiquitous expression pattern from early embryonic up to adult stages. Its near-ubiquitous expression raised early speculation that it may have a “general housekeeping role” [10]. The phenotype of PPAR*β*-null mice highlights its role in development. PPAR*β* deletion induces a high rate of embryonic mortality around early embryonic day 10.5 (E10.5) due to a placental defect [7, 11, 12]. The phenotype of the surviving PPAR*β*-null mice is rather mild. They present a reduction of adipose tissue [7], an altered skin inflammatory response [7, 13], a decreased number of Paneth cells in the intestine [14], some discrete metabolic modification in muscle [7, 15], and impaired wound healing [16]. PPAR*β*-null mice also present a myelin alteration [7] but exploration of the PPAR*β* function in the brain remains sketchy. In this review, we highlight the few known facts and propose some hypotheses.

## 2. EVIDENCE FOR PPAR*β* ACTIVITY IN THE BRAIN

**Figure 1:**
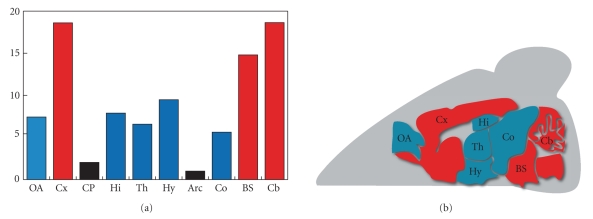
* Expression of PPAR*β* in the adult mouse brain*. Quantitative and spatial expression of PPAR*β* in the mouse brain: (a) Quantitative RT-PCR data, (b) Brain sagittal section. Moderated expression levels are in red and weak expression in blue. OA: olfactory areas, Cx: cerebral cortex, CP: caudate putamen, Hi: hippocampus, Th: thalamus, Hy: hypothalamus, Arc: arcuate nucleus, Co: colliculus, BS: brain stem, Cb: cerebellum. (MousePat: http://www-mci.u-strasbg.fr/mousepat/-consulted 8-08-2008).

During development, PPAR*β* expression starts at mid-gestation, around E10.5 days in rats, and then reaches a peak in all neural tissue between E13.5 and E15.5 [17]. Even though it then fades slightly, it remains high all through development and adult life. In the adult brain, PPAR*β* is expressed ubiquitously, with high levels found in the cerebral cortex, thalamus, cerebellum, and brain stem [4, 18, 19] (Figure 1).

Most brain cell types appear to express PPAR*β*. Immunostaining, western blots, and RT-PCR confirmed their expression in primary cultures of embryonic cortical neurons [20]. Analyses of adult brain sections enabled more detailed observations [21] revealing that pyramidal cells of the cerebral cortex, neurons of the hypothalamus, and accumbens nuclei show high PPAR*β* expression. In situ hybridization coupled to immunolocalization revealed PPAR*β* mRNA and protein expression within the oligodendrocytes of the corpus callosum [19, 22]. PPAR*β* is also expressed in primary cultures of rat cortical and cerebral astrocytes, as well as in mouse cortical astrocytes [23, 24], even if, in vivo, astrocytes appear negative to PPAR*β* immunostaining, at least in the hippocampal commissure [19, 22].

Thus, the expression of PPAR*β* is documented in the three main neural cell types: neurons, astrocytes, and oligodendrocytes, whereas we still have no information for microglia cells. Interestingly, PPAR*β* mRNA is also expressed in the rat brain capillary endothelial cells [25], suggesting that it plays a role in the brain-blood barrier.

Concerning its cellular localization, PPAR*β* immunostaining is detected in the cytoplasm and neurites of some neurons [21], raising the question of PPAR*β* nongenomic effects in these specific cells. However, its main localization is nuclear, as revealed by its exclusive detection in the nuclear fraction of whole brain protein extracts [26].

PPAR*β* expression pattern suggests that it is involved in basic physiological functions in the brain. However, the brain phenotype of PPAR*β*-null mice is poorly documented. In one study, the authors noted that PPAR*β*-null mice brain diameters are significantly smaller than in wild-type mice, most likely due to their relatively smaller body size [7]. Histological examination revealed alterations in the extent of myelination in the corpus callosum, more often in female than in male mice (three of five females; two of seven males). This defect is absent in other parts of the brain, including the cerebellum and brain stem. The two main proteins playing a role in myelin organization, myelin basic protein (MBP) and proteolipid protein (PLP), are not differentially expressed in the corpus callosum of PPAR*β*-null mice, despite a putative PPRE in the PLP promoter [27].

Thus, the full functional exploration of PPAR*β* activity in the brain remains to be performed. In the following sections, we summarize and comment on studies that chart the first leads in this domain.

## 3. PPAR*β* AND LIPID METABOLISM IN THE BRAIN

The best-known role of PPAR*β*, with possible consequences on the whole organism, is to increase lipid oxidative metabolism in muscles, in particular fatty acid peroxisomal-*β* oxidation [28]. Along this line, long-term treatment of obese animals with the PPAR*β* agonist GW501516 causes significant weight loss accompanied by improvement of the lipoprotein profiles and metabolic parameters [29, 30]. Interestingly, the brain lipid content of PPAR*β*-null mice is altered in females: they present a 24% and 17% increase in plasmenylethanolamine and phosphatidylserine, respectively, and a 9% decrease in the level of phosphatidylinositol when compared to controls animals [31]. The altered phospholipid composition in female PPAR*β*-null brains could result from a defect in brain peroxisomal acyl-CoA utilization. If true, this could explain the altered myelination observed in PPAR*β*-null mice, as inactivation of peroxisomal *β* oxidation function induces demyelination in human and mouse brain [32]. The fact that a PPAR*β* selective agonist (L165041) increases the expression of AcylCoA synthtase 2 (ASC2) in rat brain cell cultures [33] supports a direct role of PPAR*β* on brain lipid metabolism. ASC2 turns fatty acids into fatty acyl-CoA, a modification required for their metabolism. However, in unchallenged conditions, ASC2 expression is not changed in the adult brains of PPAR*β*-null mice compared to wild-type mice [7]. If confirmed, the modification of brain lipid composition in PPAR*β*-null mice may have multiple impacts, including a modification of membrane plasticity or an alteration of pathways requiring lipid post-translational modifications. For example, acylation is a common post-translational modification of myelin proteins [34] such as PLP, which is crucial for the stabilization of myelin sheets. Another example is the processing of Shh, which undergoes cholesterol addition and palmitoylation to contribute to forebrain patterning [35]. Finally, an alteration of the lipid metabolism can directly perturb neural differentiation, as discussed above.

Numerous recent papers have highlighted the role of PPAR*β* in cholesterol metabolism [36–40]. Even though the CNS accounts for only 2.1% of body weight, it contains 23% of the sterols present in the whole body pool. In PPAR*β*-null mice, the total cholesterol content of the brain is not changed compared to that of wild-type mice [31]. Nevertheless, it does not imply that brain cholesterol metabolism is not impaired. Cholesterol in the CNS comes almost entirely from in situ synthesis with little or no transfer from the blood into the brain, whereas cholesterol can leave the brain and pass into the general circulation in the form of 24-hydroxycholesterol. Inside the brain, a large amount of the cholesterol turnover is between glial cells and neurons during CNS development, but also occurs in the context of neuronal repair and remodeling. This internal recycling involves the cellular exchange of cholesterol through intermediate binding to apolipoproteins E and A1. Interestingly, alteration of the cholesterol balance across the whole body may alter sterol recycling and apolipoprotein E expression within CNS, thereby affecting neuron and myelin integrity [41]. Altogether, these observations are a strong incitement to exploring whether PPAR*β* acts on the fatty acids and cholesterol metabolisms in the brain.

## 4. PPAR*β* AND NEURAL CELL FATE

**Figure 2:**
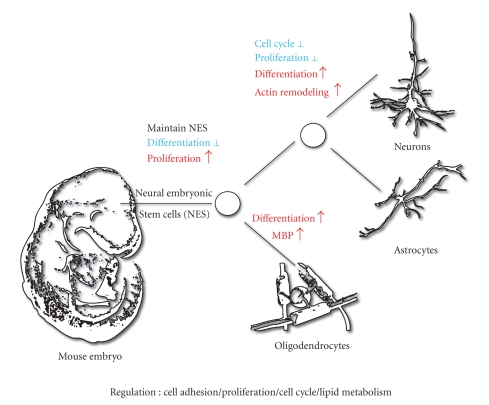
*PPAR*β* potential role in neural cell differentiation during development.* PPAR*β* is expressed in the embryo and in the differentiating cells of the CNS and plays important role for the maintenance and/or differentiation of neural stem cells (NSC). PPAR*β* maintains NSC in an undifferentiated proliferative status [45]. Once the NSC cells have started to differentiate, PPAR*β* could play a role in (1) neuronal differentiation, inducing the morphological characteristics and the gene expression patterns of neuronal cells, (2) promoting the oligodendrocyte precursor cells (OPC) differentiation [46], strongly expressing PPAR*β* [47], to mature oligodendrocytes [7]. In promoting differentiation, PPAR*β* also influences cell cycle and proliferation rate. Up to today no role has been clearly established for PPAR*β* in astrocyte differentiation. (↑ Activation in red, ⊥ Repression in blue).

In different models, PPAR*β* has a prodifferentiation activity, observed for various cell types such astrophoblast giant cells [12], adipocytes [42, 43], sebocytes, Paneth cells in the intestine [14], and keratinocytes under normal and inflammatory conditions [13, 44]. There is now some evidence that PPAR*β* favors neural cell differentiation (Figure 2). However, observations vary according to the models investigated and many questions must be further addressed.

Oligodendrocytes are the myelin-producing cells in the CNS. The timing of oligodendrocyte differentiation depends on an intrinsic clock in oligodendrocyte precursor cells (OPC) that counts time or cell divisions and limits precursor cell proliferation. The timing of oligodendrocyte differentiation depends on hormonal signals such as thyroid hormones, glucocorticoids, and retinoic acid, which bind and activate their cognate nuclear receptor [48]. Two facts suggest a role of PPAR*β* in OPC differentiation: first, the strong expression of PPAR*β* in these cells [47], and second the partial alteration of the corpus callosum myelination in the brain of PPAR*β*-null mice [7]. Cell culture experiments support this hypothesis. In primary glial cell cultures and oligodendrocyte enriched cultures prepared from neonatal mouse brains, different PPAR*β* agonists accelerated OPC differentiation within 24 hours [46]. These treatments induced by two- to three-fold the number of oligodendrocytes with processes and huge membrane sheets. They also increased the expression of some differentiation markers, such as MBP and PLP, at the mRNA and protein levels [46]. While this prodifferentiation activity remains to be further documented in vivo, it suggests that PPAR*β* contributes to the dietary lipid activity in accelerating myelinogenesis [49, 50].

At the present time, we have few clues for how PPAR*β* acts on oligodendrocyte differentiation while not affecting oligodendrocyte precursor proliferation [46]. Oligodendrocytes synthesize myelin and thus are the major lipid producing cells in the CNS. Interestingly, a majority of the cells that are sensitive to PPAR*β* during their differentiation (adipocytes, trophoblast giant cells, sebocytes, and keratinocytes) are characterized by lipid accumulation during differentiation. Indeed, disruption of PPAR*β* resulted in an alteration of mouse adipose tissue development [7]. In 3T3-L1 and 3T3-F442A cell lines which replicate in vitro adipocyte differentiation, PPAR*β* is one of the early activated genes [51]. In contrast to this early implication in adipocyte differentiation, PPAR*β* regulates the late stages of sebaceous cell differentiation [52]. It is also the most effective PPAR isotype in stimulating lipid accumulation in keratinocytes [53]. Finally, the differentiation of giant cells in mouse placenta is accompanied by a PPAR*β*-dependent accumulation of lipid droplets and an increased expression of the adipose differentiation-related protein (ADRP, also called adipophilin), which may participate in lipid metabolism and/or steroidogenesis [12]. While the specificity of each of these contexts suggests that PPAR*β* acts on different programs of differentiation [54], this contribution of PPAR*β* to lipid synthesis may well also apply in oligodendrocytes, accelerating their differentiation from precursor to fully mature cells.

PPAR*β* action on neuronal differentiation is still under investigation. In primary cultures of embryonic cortical neurons, PPAR*β* is expressed in the neuron nuclei and increases in relation to the degree of maturation of these cells, in correlation with its heterodimer partners RXR *β* and *γ*. The concomitant induction of the PPAR*β* target gene ACS2 suggests that PPAR*β* is activated [20]. With respect to neuronal differentiation per se, the available data are limited to human neuroblastoma cell lines. Exposure of these PPAR*β* expressing cells to PPAR*β* agonists, either oleic acid or the GW610742X, triggers neuronal differentiation [20], characterized by neuritis outgrowth. Both compounds also promote morphological modifications of the actin filaments [55] and induce the expression of a series of neuronal differentiation markers such as growth associated protein 43 (GAP-43), neural cell adhesion molecule (N-CAM), and neurofilament-200 [55]. As the cells undergo differentiation, their proliferation rate, cellular migration, and invasiveness are slowed down by oleic acid or GW610742X treatments. In parallel, oleic acid or the GW610742X agonist increases the expression of the cyclin inhibitor p16, indicating that PPAR*β* activation may be able to promote cell cycle arrest [55]. All GW610742X effects are related to PPAR*β*, as demonstrated by the use of siRNA silencing. In contrast, the oleic acid effects were never fully reverted thereby indicating that they are only partially mediated by PPAR*β*.

These observations however need to be confirmed in more physiological models, such as primary cultures of neurons and mouse models. In fact, they contrast with other data suggesting that PPAR*β* rather maintains neural stem cells in an undifferentiated, proliferative status [56]. In the model of neurospheres cultures, prepared from the periventricular tissue of the adult mouse brain, western-blot and RT-PCR analysis demonstrated that PPAR*β* is expressed in undifferentiated neurospheres (S0) and decreases in differentiated neurospheres (S10) [45]. In line with this, the expression of PPAR*β* in primary cultures of mouse cortical astrocytes also decreases between 14 and 21 divisions, possibly in relation to the decreased astrocyte proliferation at confluence [45]. In these studies, PPAR*β* activity correlates with the expression of genes involved in cell cycle [20, 45, 57]. Nevertheless, PPAR*β* action on cell proliferation is highly dependent on the cell type. For example, it exerts a proproliferative action on preadipocytes [58, 59] and an antiproliferative action on keratinocytes [60]. Moreover, PPAR*β* proproliferative activity on neural stem cells does not necessarily exclude a prodifferentiation activity.

After this tour of the possible functions of PPAR*β* in the brain, ranging from metabolism to neural cell fate, the next sections of this review highlight the consequent roles of PPAR*β* in brain alterations and repair.

## 5. PPAR*β* IN BRAIN ISCHEMIA

The role of PPAR*β* in brain repair was first addressed in a model of focal cerebral ischemia, with a middle cerebral artery occlusion. Compared with wild type, PPAR*β*-null mice exhibited a significant increase in the infarct size [61, 62], suggesting that PPAR*β* exerts a neuroprotective activity. Intriguingly, the difference in infarct size between wild-type and PPAR*β*-mutant mice was detected by RMN as early as 30 minutes after performing the ischemia [62], suggesting that PPAR*β* plays a role in the very early events. Reciprocally, in a transient middle cerebral artery occlusion, intracerebral infusion of L-165041 or GW501516 in rat ventricle significantly attenuated the ischemic brain infarct size 24 hours after reperfusion [63]. Several hypotheses concerning the molecular mechanism of this neuroprotective activity are discussed below.

An important activity of PPAR*β* is to promote cell survival under stress conditions, as demonstrated in keratinocytes during skin wound healing [50] and in primary keratinocyte exposed to inflammatory signals [64]. PPAR*β* activation also promotes renal cell survival following hypertonic stress [65] as well as oxidative stress [66]. Neural cells also seem to be sensitive to PPAR*β* activation under stress conditions. In primary cultures of rat cerebellar granule neurons, treatment with GW0742 significantly reduced cell death during a 12-hour exposure to low-KCl media. However, prolonged incubation (48 hours) with GW0742 produced significant inherent toxicity [67]. In a different context, human neuroblastoma SH-SY5Y cells were exposed to a variety of chemicals provoking cell death, such as thapsigarginand the endoplasmic reticulum calcium ATPase inhibitor, 1-methyl-4-phenylpyridinium. Treatment with two PPAR*β* agonists, L-165041 or GW501516, significantly attenuated cell death in a concentration-dependent manner [63]. In vivo, in a model of middle cerebral artery occlusion, an increase of malondialdehyde and a decrease of glutathione and manganese superoxide dismutase in PPAR*β*-null mice argue for increased brain oxidative stress. This phenotype was associated with a relative increase in interferon *γ* but a lack of TNF*α* production [61]. Thus, PPAR*β* could regulate central inflammation and antioxidant mechanisms in the damaged brain. Some known PPAR*β*-regulated genes could explain these observations, including COX2 [68], which promotes inflammatory reactions by prostaglandins synthesis [69]. Nevertheless, in vivo or in vitro treatment of T cells with GW0742, a PPAR*β* selective agonist, did not reduce IFN*γ* production [70]. Alternatively, PPAR*β* could also act via the regulation of IL-1*β* to reduce astroglial and microglial inflammatory activation, as suggested in experimental autoimmune encephalomyelitis (EAE) [70]. PPAR*β* inflammatory response could also involve a direct interaction between PPAR*β* and the inflammatory suppressor protein, BCL-6, as in macrophages [71].

While we can reasonably hypothesize that PPAR*β* may indeed play a role in modulating inflammation and controlling oxidative damages, thereby contributing to moderate ischemia lesion, other hypotheses may also contribute to understanding the increased ischemic lesion in PPAR*β*-null mice. An interesting one concerns the role of PPAR*β* in the vascular system. For example, a different patterning of vascular territories would result in a different infarct size occurring at the very first time point postischemia. An experiment designed to visualize the vascular tree would then provide an important control. Local conditions of blood flow might also affect the outcome of an ischemia experience. However, no data so far have been published concerning hemodynamic parameters in the brain of PPAR*β*-null mice. Finally, angiogenesis itself might be concerned. This is supported by investigations performed on an unrelated model, using subcutaneous inoculation of lung carcinoma cells carrying the two PPAR*β* wild-type alleles in a PPAR*β*-null mutant mouse. In this model, the tumor growth was impaired, due to the absence of PPAR*β* in the stroma cells surrounding the developing tumor. This led to a diminished blood flow and a reduced development of hyperplastic microvascular structures [72]. Thus, PPAR*β* deletion could also affect the delayed response to ischemia by impairing angiogenesis [73].

An interesting cellular property that may be overlooked in the search for functional disturbances linking PPAR*β* and cerebral ischemia is cell-cell adhesion and matrix-cell adhesion. As proposed by del Zoppo et al. [74], matrix cell adhesion receptors might be essential for the maintenance of the integrity of the blood-brain permeability barrier, challenged upon local injury. In particular, focal ischemia suddenly alters the matrix constituents and changes the expression of cell adhesion receptors, locally increasing vascular permeability [75]. We summarize below some indirect evidence for a role of PPAR*β* in cell adhesion, which may contribute to its neuroprotective activity.

In an in vitro study, modulation of PPAR*β* activity in F9 teratocarcinoma cells positively correlated with modulation of neural cell adhesion molecule (NCAM) expression. In fact, F9 cells treated with valproic acid increased the expression of PPAR*β*, but not that of PPAR*α* or PPAR*γ*, while also enhancing the expression of cell adhesion molecules such as NCAM and PST1. Reciprocally, overexpression of a dominant-negative PPAR*β* reduced the NCAM induction [76]. In endothelial cells of human umbilical, PPAR*β* directly regulated a few key cell-adhesion genes. PPAR*β* agonists GW0742 and GW501516 significantly inhibited TNF*α* induced expression of vascular cell adhesion molecule-1 and E-selectin, and the ensuing endothelial-leukocyte adhesion [77]. Chromatin immunoprecipitation assays showed that GW0742 switched the interaction of BCL-6, a transcription repressor, from PPAR*β* to the vascular cell adhesion molecule-1 (VCAM-1) gene promoter. Evidence for a role of PPAR*β* in cell adhesion/migration also stems from PPAR*β* activity in wound healing, where it regulates the intracellular pathways activated during keratinocyte directional sensing, polarization, and migration [16]. In these events, redistribution of integrins requires Akt1 activity while NF-*κ*B stimulates the production of the metalloproteinase MMP-9, which allows the digestion of extracellular matrix, a process required for cell migration. Since PPAR*β* concomitantly regulates both Akt1 and NF-*κ*B [64], it would be of great interest to study these two pathways in the damaged brain.

In a more general way, cell adhesion is a key developmental process, as it determines the location of a cell by regulating its capacity to move in a tissue or to be restricted to a defined area. It is also crucial for neural cell-cell interactions, including axon guidance and synapse formation, processes tightly connected to the functioning of the brain itself, such as learning and memory. Taking these observations together points to the particular need for a better understanding of PPAR*β* action on neural cell adhesion.

## 6. USING PPAR*β* AGONIST/ANTAGONIST TO TREAT BRAIN DISEASES

Whereas many studies have explored the possible benefits of targeting the ever popular PPAR*γ* isotype with regard to its neuroprotective effect [78], an interest in the more ubiquitous PPAR*β* isotype is recently emerging. In this last section, we will therefore review studies that have explored PPAR*β* targeted therapeutics for a variety of brain diseases.

Experimental autoimmune encephalomyelitis (EAE) [79, 80] is a T-cell mediated autoimmune disease that involves inflammatory activation of brain glial cells, and is used as a model for multiple sclerosis. Oral administration of PPAR*γ* agonists reduces the incidence and severity of clinical, histological, and biochemical symptoms in EAE. The GW0742 PPAR*β* agonist has also beneficial effects, demonstrated in a mouse model of EAE, in which mice were immunized with an encephalitogenic myelin oligodendrocyte glycoprotein (MOG) peptide [70]. When given at the time of immunization, GW0742 had only a moderate effect on the appearance and severity of clinical symptoms. Nevertheless, prolonged treatment of mice already exhibiting signs of the disease improved their clinical status. Intriguingly, the clinical improvement of cortical lesions contrasts with no significant reduction of the cerebellar lesions. The mechanism does not involve T cell activation, oligodendrocyte maturation, or survival, but probably a reduction of astrocyte and microglial inflammatory responses [70]. Thus, PPAR*β* and PPAR*γ* agonists differ both in the timing of treatment efficiency and the molecular mechanism involved [81].

The absence of PPAR*β* agonist action on oligodendrocyte maturation or survival in this model of EAE is surprising (see Section 4). It is thus tempting to explore how the PPAR*β* agonist could alter the course of a nonautoimmune demyelinating disease, such as a diabetes complication or leukodystrophy. In particular, adrenoleukodystrophy is a rare inherited disorder that leads to progressive failure of the adrenal gland, brain damage, and eventually death. In this disease, the mutation of the ATP-binding cassette subfamily D member 1 (ABCD1) gene leads to a reduction of beta oxidation in peroxysomes with the accumulation of very long chain fatty acids in the adrenal cortex and brain, causing a progressive inflammatory demyelination. Because of its crucial role in peroxisome proliferation and fatty oxidation, PPAR*α* was the prime target tested in a therapeutic approach. However, no direct effect of PPAR*α* agonists could be seen in modulating ABCD2, the closest relative of ABCD1, in the brain [82]. It would still be interesting to test a PPAR*β* agonist because of its combined role in fatty acid oxidation and in oligodendrocyte maturation.

Alzheimer disease is a neurodegenerative disorder characterized by cognitive and memory deterioration, progressive impairment of activities, and a multiplicity of behavioral and psychological disturbances. While not fully understood, the mechanism of the alteration includes an extracellular accumulation of amyloidal plaque formed by oligomerisation of the amyloidogenic peptide A*β* 1–42, and the accumulation of the Tau protein responsible for neurofibrillar degeneration. Interestingly, the noradrenalin (NA) neurotransmitter protects neurons from inflammation [83], via a mechanism that is partially PPAR*β* dependent. In fact, in the model of primary cultures of rat cortical neurons exposed to oligomeric amyloid beta, NA partially reduced neuronal damage and toxicity, as assessed by a reduction in the release of LDH. Interestingly, there was a concomitant two fold induction of PPAR*β* mRNA and protein levels. The NA neuroprotective effects were partially blocked by cotreatment with a PPAR*β* selective antagonist. Moreover, the selective PPAR*β* agonist GW742 reduced LDH release to the same extent as did the NA, suggesting that PPAR*β* is the main mediator of NA action [84]. Nevertheless, high concentrations of GW742 (50 *μ*M) are required in order to observe this effect [85], and further in vivo studies are required to evaluate the true potential of PPAR*β* agonists as therapeutic tools for Alzheimer disease.

Finally, PPAR*β* agonist treatment could be beneficial in another notorious neurodegenerative disorder: Parkinson disease, characterized by the disappearance of the dopaminergique neurons with alteration of the nigrostriatal pathways. Beside the classic occurrence of Parkinson disease, whose etiology is mainly unknown, the synthetic opiate 1-methyl-4-phenyl-1,2,3,6-tetrahydrodropyridine (MPTP) causes Parkinsonism in young drug-addicted individuals. Iwashita et al. showed that an L-165041or GW501516 intracerebroventricular infusion 48 hours before the first injection of MPTP protects against depletion of striatal dopamine and its metabolites [63].

These different studies highlight the need to characterize and optimize some PPAR*β* agonists for their capacity to cross the blood-brain barrier in order to treat various acute and chronic brain disorders.

## 7. CONCLUSION

This review highlights the few data available on PPAR*β* activity in the brain. While sometimes highly speculative, it underlines the good reasons to pursue dedicated research in this domain. The brain is the second most lipid-enriched tissue after adipose tissue. Products of fat metabolism, free-fatty acids, ketone bodies, and glycerol dominate metabolic pools in early development, as a consequence of the milk diet. The high expression of the PPAR*β* lipid sensor during brain maturation suggests that it is a key regulator of brain metabolism during neurodevelopment. Moreover, PPAR*β* anti-inflammatory and prosurvival activities may play a prominent role in the acute phase of brain injury. In addition, PPAR*β* prodifferentiation activity, in particular on oligodendrocyte lineage, is of potential benefit in the treatment of neurodegenerative diseases. Therefore, in view of its potentially wide therapeutical use, it appears crucial to carry out in depth studies of the basic mechanism of PPAR*β* activity, in order to understand the molecular network driven by this receptor in the brain.
